# Home Fortification of Complementary Foods Reduces Anemia and Diarrhea among Children Aged 6–18 Months in Bihar, India: A Large-Scale Effectiveness Trial

**DOI:** 10.1093/jn/nxab065

**Published:** 2021-04-20

**Authors:** Melissa F Young, Rukshan V Mehta, Lucas Gosdin, Priya Kekre, Pankaj Verma, Leila M Larson, Amy Webb Girard, Usha Ramakrishnan, Indrajit Chaudhuri, Sridhar Srikantiah, Reynaldo Martorell

**Affiliations:** Hubert Department of Global Health, Rollins School of Public Health, Emory University, Atlanta, GA, USA; Doctoral Program in Nutrition and Health Sciences Program, Laney Graduate School, Emory University, Atlanta, GA, USA; Hubert Department of Global Health, Rollins School of Public Health, Emory University, Atlanta, GA, USA; Doctoral Program in Nutrition and Health Sciences Program, Laney Graduate School, Emory University, Atlanta, GA, USA; Hubert Department of Global Health, Rollins School of Public Health, Emory University, Atlanta, GA, USA; Doctoral Program in Nutrition and Health Sciences Program, Laney Graduate School, Emory University, Atlanta, GA, USA; Hubert Department of Global Health, Rollins School of Public Health, Emory University, Atlanta, GA, USA; CARE India, Patna, India; Arnold School of Public Health, Department of Health Promotion, Education, and Behavior, University of South Carolina, Columbia, SC, USA; Hubert Department of Global Health, Rollins School of Public Health, Emory University, Atlanta, GA, USA; Doctoral Program in Nutrition and Health Sciences Program, Laney Graduate School, Emory University, Atlanta, GA, USA; Hubert Department of Global Health, Rollins School of Public Health, Emory University, Atlanta, GA, USA; Doctoral Program in Nutrition and Health Sciences Program, Laney Graduate School, Emory University, Atlanta, GA, USA; CARE India, Patna, India; Project Concern International, Delhi, India; CARE India, Patna, India; Hubert Department of Global Health, Rollins School of Public Health, Emory University, Atlanta, GA, USA; Doctoral Program in Nutrition and Health Sciences Program, Laney Graduate School, Emory University, Atlanta, GA, USA

**Keywords:** anemia, multiple micronutrient powders, India, children, hemoglobin

## Abstract

**Background:**

Home fortification of complementary foods with multiple micronutrient powders (MNPs) is recommended to reduce child anemia in resource-poor settings. However, evidence of program effectiveness in India to guide policies and programs is lacking.

**Objectives:**

We implemented a large-scale intervention of MNPs in Bihar, India. The primary outcome was MNP consumption and change in hemoglobin concentration among children aged 6–18 mo between baseline and endline (12 mo). Secondary outcomes were change in child weight and length and infant and young child feeding (IYCF) practices (initiation, diversity, and feeding frequency). Ad hoc analyses included changes in anemia; stunting; underweight; wasting; and reported diarrhea, fever, and hospitalization.

**Methods:**

We conducted a cluster-randomized, effectiveness trial in >4000 children within the context of ongoing health and nutrition programs implemented by CARE, India. Seventy health subcenters were randomly assigned to receive either MNPs with IYCF counseling (intervention) or IYCF counseling only (control). We used an adjusted difference-in-difference approach using repeat cross-sectional surveys at baseline and endline to evaluate impact.

**Results:**

At baseline, 75% of intervention and 69% of control children were anemic and 33% were stunted. By endline, 70% of intervention households reported their child had ever consumed MNPs, and of those, 64% had consumed MNPs in the past month. Relative to control, hemoglobin concentration increased (0.22 g/dL; 95% CI: 0.00, 0.44 g/dL) and anemia declined by 7.1 percentage points (pp) (95% CI: –13.5, –0.7 pp). There was no impact on anthropometry nor IYCF practices. However, there was a decline of 8.0 pp (95% CI: –14.9, –1.1 pp) in stunting among children aged 12–18 mo. Diarrhea prevalence in the past 2 wk was reduced by 4.0 pp (95% CI: –7.6, –0.4 pp).

**Conclusions:**

Home fortification of complementary foods within a government-run program in Bihar had moderate compliance and caused modest improvements in hemoglobin and reductions in anemia and diarrhea prevalence.

## Introduction

Home fortification of complementary foods with multiple micronutrient powders (MNPs) is an evidence-based strategy recommended to reduce child anemia in resource-poor settings (1–3). MNPs are provided as a dry powder in single-serving sachets that may be sprinkled once daily over food before consumption. MNPs are available in different formulations containing encapsulated iron, vitamin A, and zinc, among other vitamins and minerals (4). Given the strength of the evidence base, largely from efficacy studies, WHO released strong recommendations in 2011 in support of home fortification of foods with MNPs to improve iron status and reduce anemia among young children (5). Subsequently, public health programs supporting home fortification with MNPs among infants and children aged <2 y have expanded rapidly worldwide (6, 7). As of 2020, 32 countries in Asia, 15 in Africa, and 12 in Latin America, with 10 national and 32 subnational programs, actively implemented MNP programs (8). In a recent systematic review of the literature, home fortification with MNPs reduced anemia in young children by 18% and iron deficiency by 53% (3).

Whereas there is a strong evidence base for the efficacy of MNPs on anemia, the evidence is less clear for large-scale effectiveness evaluations (3, 9). A recent review of 9 effectiveness studies found that children who received MNPs had a significantly lower risk of anemia (RR: 0.89) but indicated no significant effect on hemoglobin (Hb) concentration. The effectiveness of MNPs on nutritional biomarkers and functional outcomes in children is limited by few well-designed and -powered studies (10). Effectiveness trials are important because they help improve understanding of how best to increase uptake and impact under real-world conditions. Many factors can hinder MNP program implementation, ranging from supply side factors such as stock-outs to demand side issues including low adoption and poor adherence to dose regimens (11).

Concerns about potential adverse effects of MNPs on child illness have also been raised (12). In a systematic review of efficacy and effectiveness studies, Tam et al. (10) found MNP supplementation was associated with a 30% increased risk of diarrhea (10). On the other hand, after reviewing the evidence, Suchdev and coauthors (3) concluded that the benefits of MNPs outweigh potential risks. The benefits of MNPs may be particularly pronounced in settings of high anemia such as South Asia, which has the largest global burden of anemia with >102 million anemic children (13). Within South Asia, Bihar, India, has among the highest prevalence of anemia, affecting 63.5% of children aged 6–59 mo (14). Diet may be a critical contributor. Only 23% of children aged <2 y meet basic infant and young child feeding criteria such as minimum meal frequency, minimum dietary diversity, and minimum adequate diet, placing these children at higher risk for micronutrient deficiencies (15). To address the high burden of anemia and suboptimal feeding practices, CARE, in collaboration with the state government of Bihar and Emory University, identified a need for testing innovative nutrition solutions that have the potential to scale. One of these potential solutions was a program designed to integrate delivery of MNPs with the promotion of age-appropriate infant and young child feeding practices (IYCFs). Here, we present the results of a 1-y effectiveness evaluation of a home fortification of complementary foods program on child nutritional status, feeding practices, and illness conducted in Bihar. This study was designed to address the gap in our knowledge of the feasibility and effectiveness of MNP programs within the local context and using existing government frontline workers (FLWs). The program was delivered to >10,000 children and is the largest effectiveness study of MNPs carried out to date.

## Methods

The home fortification of complementary foods study was conducted in the West Champaran district of Bihar among children aged 6–18 mo and their caregivers (clinical trial registration: NCT02593136). The study was implemented between January and December 2015 and nested in the context of the larger Integrated Family Health Initiative program led by a consortium of partners, including Emory University, and implemented by CARE India and the state government. The evaluation used a repeated cross-sectional, cluster-randomized, community trial design. Caregivers of children aged 6–18 mo in the catchment of each health subcenter (HSC) received either MNPs and IYCF counseling (intervention) or IYCF counseling alone (control) for 12 mo. The program reached over 10,000 families in Bihar.

Multiple micronutrient powder sachets were purchased from DSM Nutraceuticals after extensive formative research was conducted to inform the development of contextualized packaging and messages. The local product name was Jeevan Jyoti (Light of Life), which was promoted with the slogan, “One packet a day, whether it's a boy or girl.” Messages on MNP use were integrated with counseling materials on IYCF. Each MNP sachet contained 12.5 mg iron (ferrous fumarate), 5 mg zinc (zinc gluconate), 0.16 mg folic acid, 0.3 mg vitamin A (vitamin A acetate), 30 mg vitamin C (ascorbic acid), 0.9 μg vitamin B-12, and 90 μg iodine, coated in a maltodextrin base.

The program design has been described in detail elsewhere (16, 17). Briefly, in both intervention and control communities, CARE worked closely with government officials on strengthening the health system and capacity-building of FLWs. In all communities, FLWs were provided enhanced training and job aids on IYCF. Pamphlets with visual aids on age-appropriate IYCF practices were provided to FLWs to use with and provide to families (17). Pamphlets were developed from and informed by formative research and existing visual aids and were designed to demonstrate feeding quantity, frequency, ideal consistency of complementary foods, and hygiene-related details including handwashing for both caregivers and infants prior to and after feeding (17).

In intervention communities, in addition to IYCF counseling, government Health and Integrated Child Development Services FLWs, including Accredited Social Health Activists (ASHAs) and Anganwadi workers (AWWs), were instructed to distribute MNPs to all children aged 6–18 mo. Auxiliary nurse midwives and lady supervisors provided supportive supervision to FLWs during program implementation. FLWs were advised to distribute 1 box of MNP (intervention group) and provide IYCF counseling (intervention and control group) to each eligible household during routine home visits with caregivers, which is an important component of the existing job profiles of ASHAs and AWWs. IYCF pamphlets and counseling for the intervention group included additional instructions on how to use MNPs and types of foods to mix them in (17). IYCF pamphlets and counseling were generally provided to the household when MNPs were first delivered, with subsequent follow-up home visits to monitor MNP use and IYCF practices and replenish MNP supplies when needed, all at no cost to the household.

FLWs were trained on proper use of MNPs and potential side effect management. FLWs were instructed to distribute up to 8 boxes (240 sachets) for each child aged 6–18 mo over the course of the year. Each box contained 30 sachets of the powder, to be used once per day for the duration of the month. Delivery of MNPs to households began in January 2015 and ended in March 2016. Children in intervention HSCs were enrolled in the program on a rolling basis as they became eligible based on age criterion. Control group HSCs did not receive MNPs at any time during the course of the study.

Primary outcome measures were changes in Hb concentration and number of MNP sachets consumed within the previous week and month. Although the total number of sachets was our registered outcome, we chose to categorize consumption because of data distributions and for ease of interpretation. Among households that reported ever receiving MNPs, consumption was categorized as any or no consumption in the past week or month. Consumption was also categorized by level of consumption (MNP sachets in past 7 d: 0 sachets, 1–5 sachets, and >5 sachets; and MNP sachets in past 30 d: 0 sachets, 1–15 sachets, and >15 sachets). Secondary outcomes included frequency of MNP sachet distribution, change in body length (operationalized as change in mean length-for-age *z* score), and change in body weight (operationalized as change in mean weight for age and weight for height). Other secondary outcomes examined complementary feeding practices, including complementary feeding for 6-mo-old children, number of daily meals within 24 h, quantity of food consumed by child per meal within 24 h, consistency of food consumed within 24 h, and diversity of foods consumed within 24 h. Additional ad hoc analyses included changes in anemia, stunting; wasting, and underweight, as well as reported diarrhea, fever, and hospitalization. These were added in response to local stakeholder interests, growing concerns of potential side effects, and for comparability with recent literature. FLW MNP distribution and program implementation and several measures of child development have been reported elsewhere (16, 17).

Due to imprecise measurement, larger samples sizes are typically needed to detect changes in behavioral outcomes than biological ones. For this reason, a priori sample sizes were calculated based on IYCF-related behavioral outcomes (minimum dietary diversity and minimum meal frequency), followed by a calculation of the subset needed to detect differences in mean Hb concentrations. To detect a 10% minimum difference in IYCF-related behavioral practices with a background prevalence of 50% at 90% power and a 2-sided 95% significance level, 2180 children were needed from each of the 2 age strata: younger children aged 6–12 m and older children aged 12–18 mo (4360 total). A subsample of 1420 children was needed from each age stratification (2840 total) to detect a 0.25 g/dL difference in Hb concentration, the primary outcome, with 90% power and a background SD of 2 g/dL. The same sample size would allow detection with 90% power of a 0.15 SD difference in length-for-age *z* scores (a secondary outcome) within each age stratum, assuming an SD of 1.2; similar estimates apply for weight and weight-for-length *z* scores. Sample size calculations were based on impact evaluation design, and we oversampled ∼5% to account for nonresponse.

The impact of the program was assessed using 2 cross-sectional surveys. Children aged 6–18 mo were selected randomly using 2-stage cluster sampling with AWCs within HSCs. Clusters were defined as HSCs selected from within 4 blocks in the study district using probability proportional to size sampling. HSCs were selected based on total population and distance to the district headquarters, excluding those prone to flooding and political instability. Out of a total of 135 HSCs in selected blocks, 70 HSCs were shortlisted by means of a simple randomization method using a random number generator (35 control, 35 intervention). Within each HSC, 5 AWCs were chosen at random; in HSCs with fewer than 5 AWCs, all AWCs were chosen. A listing of all households with children aged 6–18 mo was used to select 62 children per HSC: ∼31 children aged 6–11 mo and 31 children aged 12–18 mo. Household listings were generated by survey teams prior to the start of data collection. For Hb measurements, a subset of 20 children aged 6–11 mo and 12–18 mo were sampled per HSC. Primary inclusion criterion was child age (6–18 mo). If multiple children in a household were eligible, the oldest child was selected.

Household surveys were conducted in the local language (Hindi or Bhojpuri) at baseline (August and September 2014) and endline (February and March 2016) with caregivers of the selected children. The questionnaire was developed in English, translated, and back-translated to ensure fidelity of the questions. Data collection teams were trained, and standardized questionnaires were pretested. Standardization exercises were conducted for anthropometry and Hb concentration data collectors whereby reliability and accuracy of all length, weight, midupper arm circumference (MUAC), and HemoCue measurements were tested across teams of data collectors prior to the start of data collection. Measurements taken by data collectors were compared against those of a trained expert to ensure interrater reliability and accuracy as part of standardization protocols.

Surveys included questions on sociodemographic characteristics, IYCF practice, and MNP consumption; the Household Hunger Scale (18); and caregiver self-reported morbidity for the child in the previous 2 wk for any diarrhea and fever. Diarrhea was further classified into bloody (the presence of blood in stool), persistent (>14 d of diarrhea), or severe diarrhea (≥6 loose stools per day) during the previous 2 wk. Hospitalization refers to a child being admitted to in-patient hospitalization (>12 h). Capillary blood was collected from the heels of children aged 6–11 mo and the fingers of children aged 12–18 mo, and Hb concentrations were measured using the HemoCue 201+. Standard anthropometric procedures were used to measure child weight using the Seca 874, length using the Seca 417, and MUAC using a flexible measuring tape. Children with an Hb concentration <7 g/dL (severe anemia) or MUAC <11.5 cm (severe acute malnutrition) were referred to the nearest primary health center. HemoCue machines and Seca weighing scales were routinely calibrated in the field to ensure functionality and accuracy of measurements. Quality control measures including back-checks and spot checks were conducted on 10% of the total survey sample by independent external data quality monitors. In addition, 10% of the total survey forms were double entered to ensure data quality.

### Statistical analyses

Principal component analysis of household assets was used to create a wealth index that was subsequently divided into wealth tertiles (19). Early age at marriage was defined as marriage occurring before the caregiver was age 18 y, and maternal education was dichotomized as no education and at least some primary education. Child's age was measured in months. WHO growth standards were used to calculate weight-for-age *z* scores (WAZs), weight-for-length *z* scores (WLZs), and length-for-age *z* score (LAZs) using the WHO Anthro SAS macro (20). Standard cutoffs for stunting, wasting, and underweight were used (< –2 LAZ, < –2 WLZ, and < –2 WAZ, respectively) (21). Outliers for WAZ (< –6 or >5), WLZ (< –5 or >5), and LAZ (< –6 or >6) were excluded according to the WHO flags for biologically implausible values (22). Anemia was defined as an Hb concentration <11 g/dL. Hb values <4 g/dL or >18 g/dL were excluded as biologically implausible (23). Standard IYCF indicators were used, including timely introduction of complementary foods, minimum dietary diversity, and minimum meal frequency (24).

Means or prevalence and CIs (95% CIs) were reported. Student *t* tests with Taylor series variance were used to compare means between intervention and control groups and between baseline and endline (25). Rao–Scott chi-square goodness-of-fit tests were employed to compare categorical variables. Linear mixed-effects models were used to model the changes in the intervention group from baseline to endline relative to the control group, also called the difference-in-difference (DID). The DID approach accounts for differences at baseline and changes over time [(intervention endline – control endline) – (intervention baseline – control baseline)]. Random effects were AWCs nested within HSCs, and fixed-effects included the intervention group, survey (baseline or endline), and their product, which represents the DID. Other covariates were included as fixed effects because they differed between groups at baseline based on Student's *t* test with Taylor series variance and Rao–Scott chi-square tests. These covariates include child's age, household caste and wealth tertile, early age at marriage, and maternal education. Twenty-six observations (<0.05%) had missing values for ≥1 covariates and were excluded from the model. Mean differences (MDs) in continuous variables and prevalence differences for categorical variables with their 95% CIs were reported. Sensitivity analyses were conducted to examine effect modification based on age (6–11 mo compared with 12–18 mo). Using logistic regression models, we examined associations between consumption of MNPs (previous week and month) and anemia and Hb, respectively, within the intervention group at endline. Models examined both crude associations and associations adjusted for potential sociodemographic confounders, including age, caste, and wealth tertile. A priori α was set at *P* < 0.05 for significance. All analyses were conducted by using SAS version 9.4 (SAS Institute) using survey procedures to account for clustering of the data (26).

### Ethics

The Futures Ethics Board in New Delhi, St John's Medical College & Hospital Institutional Ethics Committee, and Emory University's Institutional Review Board approved our study procedures. Informed consent was obtained by all caregivers prior to data collection.

## Results

**FIGURE 1:**
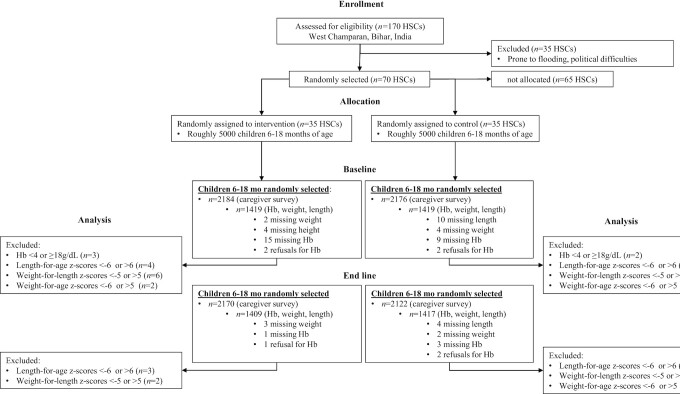
CONSORT participant flow diagram for cluster-randomized controlled effectiveness trial of multiple micronutrient powders in Bihar. CONSORT, Consolidated Standards of Reporting Trials.

We obtained data on 4360 children at baseline (2184 intervention and 2176 control) prior to the home fortification of complementary foods program launch. Hemoglobin and anthropometry were assessed in a subsample of 2805 children. After 1 y of program implementation, we obtained data from 4292 children (2170 intervention and 2122 control), including a subsample of 2819 children with hemoglobin and anthropometry. Details on sample selection and missing and excluded data are provided in **Figure 1**. Overall, <5% of the data were missing or excluded at either time point.

Household and child characteristics of participants at baseline and endline are summarized in **Table 1**. Maternal illiteracy was high, and <60% of women reported being married before age 18 y. Our sample included a range of castes, with more than half the participants identifying as belonging to other backward castes. Household hunger decreased from 9–10% at baseline to 4–5% at endline in both intervention and control communities. The differences in household hunger may be explained by seasonality. The baseline was conducted in the late monsoon season (August and September) immediately prior to the autumn harvest, months of increased food insecurity, whereas the endline was conducted in the winter months of January, February, and March following the autumn harvest (27).

**TABLE 1:** Basic household and child characteristics of participants at baseline and endline cross-sectional assessments^1^

	Baseline		Endline	
	Control (*n* = 2176)	Intervention (*n* = 2184)	Control (*n* = 2122)	Intervention (*n* = 2170)
Child age,^2^ mo	11.2 (11.1, 11.4)	11.3 (11.2, 11.5)	11.7 (11.6, 11.9)	11.7 (11.6, 11.9)
Child sex,^2^ F	50.8 (48.6, 52.9)	48.7 (46.5, 50.9)	46.6 (44.5, 48.6)	47.1 (44.9, 49.4)
Maternal illiteracy^2^	62.3 (59.8, 64.8)	57.9 (54.9, 61.0)	55.2 (52.4, 57.9)	52.0 (49.4, 54.7)
Young mother^2,3^ (aged <18 y at marriage)	65.1 (62.5, 67.7)	62.8 (60.4, 65.2)	56.6 (54.2, 59.1)	52.7 (50.3, 55.2)
Household hunger,^4^ %	9.3 (7.5, 11.1)	9.7 (7.8, 11.5)	4.6 (3.2, 5.9)	4.3 (3.3, 5.3)
Religion^2^				
Hindu	79.5 (74.8, 84.3)	78.1 (73.4, 82.8)	78.0 (73.1, 83.0)	75.7 (70.6, 80.8)
Muslim	20.5 (15.7, 25.2)	21.9 (17.2, 26.6)	22.0 (17.0, 26.9)	24.3 (19.2, 29.4)
Caste^2^				
Scheduled caste	26.4 (22.7, 30.0)	24.1 (20.5, 27.8)	21.0 (17.6, 24.5)	18.1 (14.7, 21.5)
Scheduled tribe	9.3 (6.2, 12.3)	7.3 (4.3, 10.3)	11.4 (7.8, 15.0)	7.2 (4.6, 9.8)
Other backward caste	49.9 (45.4, 53.4)	51.6 (47.6, 55.5)	56.6 (52.0, 61.1)	61.0 (56.8, 65.3)
Other	15.0 (12.4, 17.6)	17.0 (14.1, 20.0)	11.0 (8.4, 13.7)	13.7 (10.7, 16.6)
Wealth tertile^3,4,5^				
High	30.7 (27.8, 33.6)	35.9 (32.7, 39.2)	30.6 (27.7, 33.5)	36.0 (32.5, 39.6)
Middle	35.2 (33.1, 37.4)	31.5 (29.2, 33.7)	34.9 (32.6, 37.2)	31.8 (29.4, 34.2)
Low	34.1 (31.2, 36.9)	32.6 (29.6, 35.6)	34.5 (31.3, 37.7)	32.2 (28.9, 35.4)

1 Values are means or % (95% CIs). Complex survey procedures were used to account for clustering of the data.

2 Significant difference between baseline and endline (*P* < 0.05).

3 Significant difference between control and intervention group at endline (*P* < 0.05).

4 Any hunger as measured by the Household Hunger Scale.

5 Wealth index derived using principal component analysis of household assets and divided into tertiles.

Poor infant and young child feeding practices were reported in both intervention and control communities at baseline (**Table 2**). Although breastfeeding was almost universal, only ∼60% of infants met recommendations of being fed within the first hour of birth and for exclusive early breastfeeding (no prelacteal foods). A majority of infants experienced delayed introduction of complementary foods, and <40% of infants received complementary foods at the appropriate time period. Only 20% of infants received ≥4 different food groups in the past 24 h, and >80% of infants in this population failed to meet recommendations for minimally acceptable diet. Child illness and malnutrition were also very high in this population (Table 2). Approximately 12% of each group had diarrhea during the previous 2 wk, and among these, nearly 90% had persistent diarrhea (>14 d of diarrhea). Approximately 67% of the control children and 65% of the intervention children reported having a fever during the previous 2 wk. Approximately 70% of children were anemic, 42% underweight, 33% stunted, and 28% wasted. At baseline, the prevalence of anemia was lower in the control group (69%) than in the intervention group (75%).

**TABLE 2:** Child feeding practices, illness, and nutritional status among children aged 6–18 mo at baseline^1^

Child nutritional status	Control (*n* = 1406)	Intervention (*n* = 1399)
Hemoglobin, g/dL	10.2 (10.1, 10.3)	9.9 (9.8, 10.1)
Anemia,^2^ %	69.2 (66.1, 72.4)	75.3 (72.4, 78.3)
Length-for-age *z* score	–1.5 (–1.6, –1.4)	–1.5 (–1.6, –1.4)
Stunted,^3^ %	32.7 (30.0, 35.4)	33.4 (30.7, 36.1)
Weight-for-age *z* score	–1.8 (–1.9, –1.8)	–1.8 (–1.9, –1.7)
Underweight,^4^ %	41.8 (39.0, 44.5)	41.7 (39.0, 44.4)
Weight-for-length *z* score	–1.4 (–1.4, –1.3)	–1.3 (–1.4, –1.3)
Wasted,^5^ %	27.5 (24.8, 30.1)	26.5 (24.0, 29.0)
IYCF practices	Control (*n* = 2176)	Intervention (*n* = 2184)
Currently breastfeeding, %	95.7 (94.8, 96.5)	94.0 (93.0, 95.0)
Early initiation of breastfeeding (≤1 h), %	57.2 (54.1, 60.2)	56.4 (53.7, 59.1)
Avoided giving prelacteal foods, %	60.5 (57.6, 63.4)	61.7 (58.9, 64.5)
Age of initiation of complementary foods, mo	7.1 (7.0, 7.2)	7.1 (7.0, 7.2)
Timely initiation, %	39.3 (36.4, 42.3)	37.8 (35.4, 40.2)
Early initiation (<6 mo), %	5.3 (4.2, 6.4)	5.0 (4.1, 5.9)
Late initiation (>7 mo), %	55.4 (52.4, 58.3)	57.2 (54.9, 59.5)
Minimum dietary diversity,^6^ %	20.3 (18.0, 22.7)	20.3 (17.9, 22.6)
Minimum meal frequency,^7^ %	67.1 (64.5, 69.7)	64.5 (61.9, 67.1)
Minimum acceptable diet,^8^ %	16.0 (13.8, 18.2)	14.4 (12.4, 16.3)
Child illness (self-reported in past 2 wk)	Control (*n* = 2176)	Intervention (*n* = 2184)
Diarrhea,^9^ %	12.1 (10.1, 14.0)	11.6 (9.9, 13.3)
Bloody, %	7.2 (3.6, 10.8)	9.1 (5.3, 12.9)
Persistent, %	5.3 (1.9, 8.8)	8.7 (5.0, 12.4)
Severe, %	86.7 (82.4, 91.0)	89.7 (85.6, 93.8)
Hospitalization, %	1.2 (0.7, 1.8)	1.5 (0.7, 2.2)
Fever, %	67.0 (64.8, 69.3)	64.9 (62.3, 67.5)

1 Values are means or % (95% CIs). Complex survey procedures used to account for clustering of the data. IYCF, infant and young child feeding.

2 Hemoglobin concentration <11 g/dL.

3 Length-for-age *z* score < –2.

4 Weight-for-age *z* score < –2.

5 Weight-for-length *z* score < –2.

6 Four or more food groups from the Child Dietary Diversity Scale.

7 Two meals for breastfed infants aged 6–8 mo, 3 meals for breastfed children aged 9–23 mo, and 4 meals for non-breastfed children aged 6–23 mo.

8 Minimum dietary diversity and minimum meal frequency.

9 Bloody diarrhea: presence of blood in stool; persistent diarrhea: >14 d of diarrhea; severe diarrhea: ≥6 loose stools per day during the previous 2 wk.

Program reach at the household level was high, with >80% of families in the intervention group reporting having ever heard of or seen the powders at endline (**Supplemental Table 1**). Of the 70% of families that received the MNPs, 85% reported ever consuming MNPs, 63.5% of children had consumed in the past month, and 43% had consumed the powders in the past week.

A primary outcome of this trial was change in Hb concentration between baseline and endline. In the intervention group, we found a greater adjusted increase in mean Hb (0.22 g/dL; 95% CI: 0.00, 0.44 g/dL) relative to control children. Among individuals in the intervention group, Hb was 0.37 g/dL (95% CI: 0.12, 0.63 g/dL) higher in individuals who consumed ≥5 MNP sachets in the past 7 d and 0.27 g/dL (95% CI: 0.05, 0.48 g/dL) higher in individuals who consumed >15 MNP sachets during the past 30 d relative to those who consumed none (**Table 3**). Likewise, for individuals in the intervention group, consumption of ≥5 MNP sachets in the past 7 d was associated with a 36% reduction in anemia compared to those who did not consume any MNP.

**FIGURE 2:**
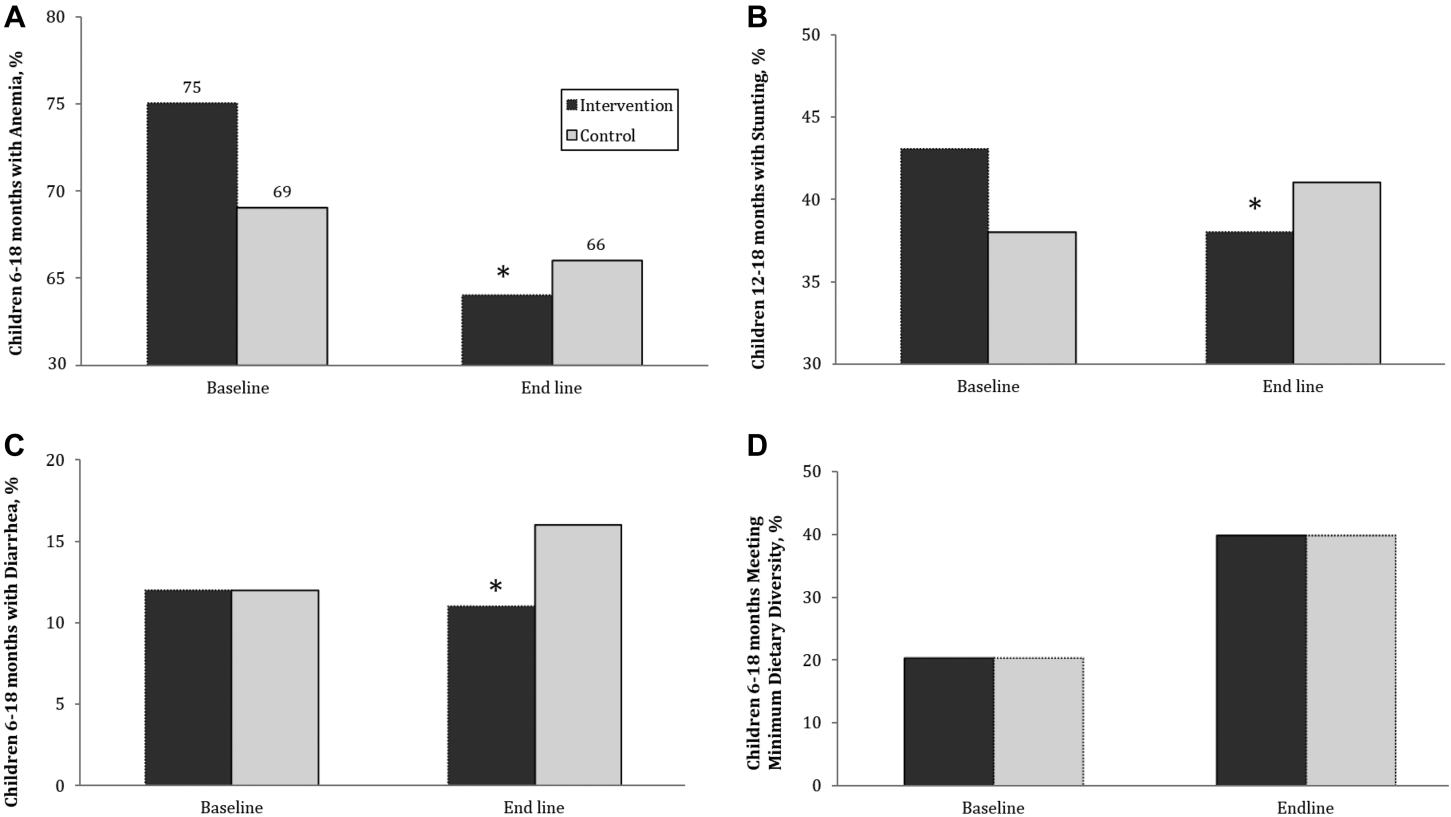
DID impact analysis comparing changes from baseline to endline in the prevalence of anemia (A), stunting (B), diarrhea in the previous 2 wk (C), and meeting minimum dietary diversity in the previous 24 h (D) in children aged 6–18 mo who received multiple micronutrient powders relative to the changes in the control group. Complex survey procedures were used to account for clustering of the data. *Different from control, *P* < 0.05. (A) DID in the prevalence of anemia among children aged 6–18 mo (7.1 pp, *P* < 0.05). (B) DID in the prevalence of stunting among children aged 12–18 mo (8.0 pp, *P* < 0.05). (C) DID in the prevalence of diarrhea in the previous 2 wk among children aged 6–18 mo (4.0 pp, *P* < 0.05). (D) DID in the prevalence of children aged 6–18 mo meeting minimum dietary diversity in the previous 24 h (no difference). DID, difference-in-difference; pp, percentage points.

For anemia, in our adjusted DID analysis, we observed a significant decrease of 7.1 percentage points (pp) in intervention communities (**Figure 2**) relative to children in control communities, representing a 9.4% decrease in prevalence (*P* = 0.001). This relation was stronger among children aged 12–18 mo, with an 8.6 pp decrease in anemia relative to control children (*P* = 0.032). We observed no measurable difference in the change in prevalence of severe anemia (Hb <7 g/dL).

In terms of secondary outcomes, there was no significant overall impact of the intervention on child anthropometry (WAZ, WLZ, and LAZ). Likewise, in ad hoc analyses, there was no significant overall impact on child stunting, underweight, or wasting. However, for older children (aged 12–18 mo), we observed an 8 pp decline in stunting among intervention children relative to controls, with the adjusted prevalence dropping from 43% to 38% in the intervention communities and increasing from 38% to 41% in control communities over the course of 1 y. We observed no impact of the intervention on the prevalence of MUAC for age, severe acute malnutrition (MUAC <11 cm), severe stunting (LAZ < –3), or severe wasting (WLZ < –3).

For secondary outcomes related to infant and young child feeding practices, several indicators improved over time, although there was no significant impact of the program on any of the child feeding practices. For example, as illustrated in Figure 2, the proportion of children meeting the requirement for minimal dietary diversity increased from 20% to 40% in both intervention and control communities. Likewise, there were increases in the proportion of children meeting recommendations for minimal meal frequency (7–8 pp improvement), minimally acceptable diet (6–7 pp improvement), and age-appropriate introduction of complementary foods (2–4 pp improvement) for all children (data not shown). Results from secondary outcomes related to FLW home visits, frequency of MNP distribution, and FLW motivation have been presented elsewhere (17).

Difference-in-difference impact analysis of the program on child anemia, nutritional status (stunting, wasting, underweight), feeding practices, and illness is shown in **Table 4**. Additional outcome indicators including those related to endline child feeding practices, illness, and nutritional status among children aged 6–18 mo are summarized in **Supplemental Table 2**. In ad hoc analyses, the prevalence of diarrhea remained stable in intervention communities but increased in the control group, resulting in a significant 4 pp reduction in the reported prevalence of any diarrhea in the past 2 wk among children aged 6–18 mo in the intervention communities relative to control children (Figure 2). This represents a decrease of 34.5%. The difference was most pronounced among children aged 12–18 mo (5 pp reduction). There were no differences in reported bloody diarrhea, severe diarrhea, or persistent diarrhea between the groups (data not shown). Likewise, there was no significant difference in reported child hospitalization or fever.

**TABLE 3:** Number of MNP sachets consumed in the previous 7 and 30 d by children in the intervention group at endline (*n* = 1407)^1^

	Crude		Adjusted^2^	
Variable	7 d	30 d	7 d	30 d
Anemia				
0 sachets	Ref	Ref	Ref	Ref
1–5^3^/1–15^4^ sachets	0.98 (0.70, 1.39)	1.03 (0.78, 1.36)	0.96 (0.68, 1.36)	1.02 (0.76, 1.35)
>5^3^/>15^4^ sachets	0.66 (0.45, 0.96)*	0.79 (0.57, 1.09)	0.64 (0.44, 0.94)*	0.78 (0.56, 1.07)
Hemoglobin concentration, g/dL				
0 sachets	Ref	Ref	Ref	Ref
1–5^3^/1–15^4^ sachets	0.15 (−0.05, 0.35)	0.10 (−0.09, 0.28)	0.19 (−0.02, 0.40)	0.13 (−0.05, 0.32)
>5^3^/>15^4^ sachets	0.35 (0.10, 0.60)**	0.23 (0.02, 0.44)*	0.37 (0.12, 0.63)**	0.27 (0.05, 0.48)*

1 Values are ORs (95% CIs) for models of anemia and β values (95% CIs) for models of hemoglobin concentration. Complex survey procedures used to account for clustering of the data. *P < 0.05; **P< 0.01. MNP, multiple micronutrient powder.

2 Adjusted for caste, wealth tertile, and age.

3 Consumption of MNP sachets in past 7 d: categorized as 0 sachets (74%), 1–5 sachets (13%), and >5 sachets (13%).

4 Consumption of MNP sachets in past 30 d: categorized as 0 sachets (68%), 1–15 sachets (14%), and >15 sachets (18%).

**TABLE 4:** Difference-in-difference analysis of the impact of home fortification of complementary foods with MNPs on child feeding practices, illness, and nutritional status of children aged 6–18 mo^1^

	Crude	Adjusted^2^	Crude	Adjusted^2^	Crude	Adjusted^2^
Variable	All children (*n* = 2836 control, *n* = 2828 intervention)		Aged 6–11 mo (*n* = 1405 control, *n* = 1403 intervention)		Aged 12–18 mo (*n* = 1431 control, *n* = 1425 intervention)	
Child nutritional status^3^						
Anemia,^4^ pp	–7.8 (–14.2, –1.4)*	–7.1 (–13.5, –0.7)*	–6.3 (–14.8, 2.1)	–5.8 (–14.3, 2.7)	–9.3 (–17.2, –1.3)*	–8.6 (–16.5, –0.8)*
Hemoglobin, g/dL	0.24 (0.02, 0.46)*	0.22 (0, 0.44)*	0.25 (–0.04, 0.54)	0.24 (–0.05, 0.53)	0.23 (–0.03, 0.49)	0.23 (–0.4, 0.49)
Stunting,^5^ pp	–2.9 (–8.1, 2.3)	–2.6 (–7.5, 2.3)	2.3 (–4.3, 8.8)	2.6 (–3.8, 9.0)	–8.4 (–15.6, –1.1)*	–8.0 (–14.9, –1.1)*
Length-for-age *z* score	0.2 (–0.1, 0.2)	0.0 (–0.1, 0.1)	–0.1 (–0.3, 0.1)	–0.1 (–0.3, 0.1)	0.0 (–0.2, 0.1)	0.1 (–0.1, 0.3)
Wasting,^6^ pp	–0.6 (–5.1, 3.8)	–0.3 (–4.7, 4.0)	–1.7 (–7.0, 3.6)	–1.6 (–7.0, 3.7)	0.3 (–6.1, 6.8)	0.3 (–5.9, 6.6)
Weight-for-length *z* score	0.1 (–0.1, 0.2)	0.1 (–0.1, 0.2)	0.1 (–0.1, 0.3)	0.1 (–0.1, 0.3)	0.0 (–0.1, 0.2)	0.0 (–0.1, 0.2)
Underweight,^7^ pp	–1.9 (–7.1, 3.3)	–1.5 (–6.3, 3.3)	–2.7 (–9.2, 3.9)	–2.3 (–8.7, 4.2)	–1.4 (–8.8, 6.1)	–1.2 (–8.3, 6.0)
Weight-for-age *z* score	0.1 (–0.1, 0.2)	0.0 (–0.1, 0.2)	0.0 (–0.1, 0.2)	0.0 (–0.1, 0.2)	0.1 (–0.1, 0.3)	0.1 (–0.1, 0.2)
	All children (*n* = 4298 control, *n* = 4354 intervention)		6–11 mo (*n* = 2118 control, *n* = 2094 intervention)		12–18 mo (*n* = 2236 control, *n* = 2204 intervention)	
IYCF practices						
Timely introduction of complementary food (at 6 mo), pp	1.6 (–3.9, 7.1)	1.7 (–3.8, 7.1)	–1.2 (–8.1, 5.8)	–0.8 (–7.7, 6.1)	4.0 (–3.0, 11.0)	4.0 (–2.9, 10.9)
Minimum dietary diversity,^8^ pp	0.0 (–4.8, 4.7)	0.2 (–4.8, 4.5)	2.9 (–3.2, 8.9)	3.0 (–3.0, 9.0)	–2.5 (–9.2, 4.1)	–2.0 (–8.5, 4.5)
Minimum meal frequency,^9^ pp	–0.8 (–5.6, 4.1)	–0.6 (–5.3, 4.2)	2.6 (–4.1, 9.3)	2.5 (–4.2, 9.2)	–3.5 (–9.4, 2.5)	–2.8 (–8.6, 3.0)
Minimum acceptable diet,^10^ pp	–0.7 (–4.8, 3.4)	1.1 (–2.9, 5.0)	3.1 (–1.3, 7.5)	3.0 (–1.4, 7.3)	–1.3 (–7.4, 4.9)	–0.7 (–6.6, 5.2)
Child illness (previous 2 wk)						
Diarrhea, pp	–4.0 (–7.7, –0.4)*	–4.0 (–7.6, –0.4)*	–3.3 (–8.0, 1.4)	–3.2 (–8.0, 1.5)	–4.7 (–9.4, 0.0)	–4.8 (–9.5, –0.1)*
Hospitalization, pp	–0.1 (–1.1, 0.8)	–0.2 (–1.1, 0.8)	0.1 (–0.9, 1.1)	0.1 (–1.0, 1.1)	–0.4 (–1.7, 1.0)	–0.4 (–1.7, 1.0)
Fever, pp	–0.8 (–5.5, 3.9)	–0.8 (–5.5, 3.8)	–3.3 (–9.6, 3.1)	–3.2 (–9.5, 3.1)	1.4 (–4.6, 7.4)	1.1 (–4.9, 7.1)

1 Values are percentage points or means (95% CIs). Complex survey procedures used to account for clustering of the data. Difference-in-difference values and 95% CIs account for differences in mean/prevalence at baseline and changes over time [(intervention endline – control endline) – (intervention baseline – control baseline)]. *P < 0.05. IYCF, infant and young child feeding; pp, percentage points.

2 Adjusted for caste, wealth tertile, age, maternal education, and early age at marriage.

3 Measured on a subsample.

4 Hemoglobin concentration <11 g/dL.

5 Length-for-age *z* score < –2.

6 Weight-for-age *z* score < –2.

7 Weight-for-length *z* score < –2.

8 Four or more food groups from the Child Dietary Diversity Scale.

9 Two meals for breastfed infants aged 6–8 mo, 3 meals for breastfed children aged 9–23 mo, and 4 meals for non-breastfed children aged 6–23 mo.

10 Minimum dietary diversity and minimum meal frequency.

## Discussion

Home fortification of complementary foods in Bihar delivered by a government program achieved moderate compliance and led to modest improvements in Hb and reductions in anemia, stunting, and diarrhea, but it did not improve child feeding practices.

Program reach was moderate, with 70% of households ever receiving MNPs, and among those, 64% of children consumed MNPs in the prior month and 43% in the past week. In other studies, coverage (defined as currently receiving or consuming MNPs or ever received/purchased or consumed) of MNPs has ranged between 22% and 93% (28). In an assessment of program bottlenecks, we found that a lack of MNP supply at the household level, in part due to infrequent distribution by FLWs, and side effects were key barriers for MNP use in our program (17). Proper counseling that can impact caregiver behaviors and perceptions has been identified as an important factor impacting fidelity and adherence to MNPs (29). Effective delivery and counseling by FLWs are essential program factors that must be considered and routinely monitored to ensure household compliance and utilization. Additional efforts are needed to build an understanding of the bottlenecks, programmatic challenges, and pathways to improve and sustain implementation, particularly when employing underpaid and overburdened government or voluntary staff (30).

The intervention increased Hb concentration (0.22 g/dL; 95% CI: 0.00, 0.44 g/dL) and reduced anemia by 7.1 pp, with greater MNP consumption and anemia declines among children aged 12–18 (8.6 pp) compared to children aged 6–11 mo. These findings are of smaller magnitude than those reported in a 2020 Cochrane review of MNP efficacy and effectiveness studies, which showed a reduction in anemia of 18% (3). Children who received MNPs had significantly higher Hb (MD: 0.274 g/dL) compared with those who did not receive the intervention or those who received placebo (3). In a systematic review by Tam et al. (10), MNP studies were divided by study design, with 32 efficacy MNP studies having a larger effect size (24% reduction in the risk of anemia) compared with 9 effectiveness studies (11% reduction in the risk of anemia) (10). Longer program exposure and delayed initiation of complementary feeding may explain the greater impact in older children. Children in the younger group of 6–12 mo at endline would have been exposed to the program anywhere from 0 to 6 mo (on average, 3 mo). In contrast, children in the older age group, 12–18 mo, would have been exposed for a longer duration, from 6 to 12 mo (on average, 9 mo). Also, the potential for impact in children aged 6–12 mo may have been limited by delayed initiation of complementary feeding, with more than half of children being introduced to complementary foods after 7 mo.

Both intervention and control communities received enhanced IYCF counseling. Although there was overall improvement in IYCF practices across both groups, there was no added benefit of MNPs on IYCF practices. Michaux et al. (31) noted that MNP interventions promote active and responsive feeding practices. Active and responsive feeding may be facilitated in part to help ensure that children eat the entire portion of food mixed with MNPs within 30 min to prevent changes in color and taste. Behavior change communication materials that provide guidance on the importance of regular meals and snacks may also promote higher meal frequency (32). However, there is mixed program evidence regarding the impact on IYCF practices. A study in Nepal found that children who consumed 30–60 MNP sachets within 3 mo were twice as likely to meet minimum meal frequency recommendations (33); similar results were also reported in Mongolia (34), Bangladesh (4), and the Kyrgyz Republic (35, 36). Locks et al. (37), in their trial of MNPs in Madagascar, found that mothers interviewed at endline had superior knowledge of feeding practices and were more likely to meet minimum dietary diversity and minimum acceptable diet requirements for their children compared to baseline (37). The authors noted that using FLWs as a delivery platform for MNPs and integrated IYCF–MNP interventions may improve IYCF practices. However, studies in Madagascar and Rwanda did not report significant improvements in IYCF practices (32, 38).

In ad hoc analyses, we noted significant declines in stunting among children aged 12–18 mo (8.0 pp) in the intervention communities. The declines in stunting among older but not younger children may also be explained by their longer duration of exposure to the intervention and MNP consumption. The zinc (5 mg) included in the MNPs may influence child growth (39, 40). Zinc supplementation has been shown to increase linear growth and weight gain modestly in previously stunted or underweight children (41, 42). Geletu et al. (43) found the prevalence of stunting to be significantly lower among children aged 9–12 mo in Ethiopia who received MNPs (43). However, recent reviews by Suchdev et al. (3) and Tam et al. (10) found no effects of MNPs on anthropometric outcomes.

We found a reduction in the prevalence of diarrhea (4.0 pp; 95% CI: –7.6, –0.4 pp) in intervention compared with control communities. Existing literature on this topic is mixed and controversial. A review by Suchdev et al. (3) reported that MNPs did not cause an increase in diarrhea, upper respiratory infections, malaria infections, or all-cause morbidity (3). A review by Tam et al. (10), on the other hand, found a 30% increased risk of diarrhea (10). Multiple potential mechanisms may explain discordant findings regarding associations between MNPs and diarrheal incidence. For example, as a result of iron supplementation, there may be a change in intestinal microflora and increase in the pathogenicity of bacteria (44). However, the zinc content of the MNPs may also have protective effects (45). Zinc supplementation has been shown to reduce diarrheal incidence by 13% (44–46). Mixed effects of the association between diarrhea and MNPs may also be attributed to context-specific factors such as diet (3). Tam et al. (10) note that increasing the dose of zinc in MNPs may further reduce risk of diarrhea in supplemented children while acknowledging competitive effects on the absorption of iron, necessitating a balance between both of these (10). Overall, there remains a need for future studies on MNPs to consolidate and standardize inconsistent reporting of side effects and morbidity, presenting an important challenge when comparing results across trials.

Important strengths of our program effectiveness design include overall reach and scale, as we were able to target upwards of 10,000 children using existing government structures and systems, including frontline staff. Our study was grounded in formative research, and program implementation was guided by a program impact pathways and data-driven management approach, which allowed for real-time monitoring and ongoing course correction (17). Limitations include the use of a cross-sectional as opposed to longitudinal design to assess impact at baseline and endline. A 0- to 6-mo cohort followed for an 18-mo period would be ideal; however, such a study design would have additional financial, logistic, and human resource costs. We were also limited by a lack of data on additional biomarkers (e.g., other micronutrients or inflammation).

Home fortification of complementary foods is a promising strategy for reduction of childhood anemia in high-burden communities, even where uptake is modest. Overall, our findings suggest that MNPs delivered to children aged 6–18 mo by a government program, employing community-based FLWs, have the potential to improve child Hb concentrations and reduce anemia, particularly among children aged 12–18 mo. In addition, secondary findings from our trial suggest MNPs improved child motor and mental development (16). The intervention had no adverse health consequences. Further work is needed to bridge implementation science domains in effectiveness trials to inform successful scale-up, address implementation barriers, and provide guidance for long-term program sustainability for home fortification.

## Supplementary Material

nxab065_Supplemental_File

## Notes

Supported by a Bill & Melinda Gates Foundation grant through a subcontract with CARE India.

Author disclosures: The authors report no conflicts of interest.

Supplemental Tables 1 and 2 are available from the “Supplementary data” link in the online posting of the article and from the same link in the online table of contents at https://academic.oup.com/jn/.

Abbreviations used: ASHA, Accredited Social Health Activist; AWC, Anganwadi center; AWW, Anganwadi worker; DID, difference-in-difference; FLW, frontline worker; HSC, health subcenter; IYCF, infant and young child feeding; LAZ, length-for-age *z* score; MD, mean difference; MNP, multiple micronutrient powder; MUAC, midupper arm circumference; pp, percentage points; WAZ, weight-for-age *z* score; WLZ, weight-for-length *z* score.
