# Supplementary material for: Home Fortification of Complementary Foods Reduces Anemia and Diarrhea among Children Aged 6–18 Months in Bihar, India: A Large-Scale Effectiveness Trial
Source: J Nutr. 2021 Apr 20;151(7):1983–92. doi: 10.1093/jn/nxab065 (PMC8245869; doi:10.1093/jn/nxab065)
Supplement: nxab065_Supplemental_File [file nxab065_supplemental_file.docx]

Home fortification of complementary foods reduced anemia and diarrhea in Bihar, India – A Large-Scale Effectiveness Trial. Melissa F. Young “Online Supplementary Material”

**Supplemental Table 1: Implementation of the multiple micronutrient powders programs among the intervention communities at end line of a cluster randomized controlled effectiveness trial of multiple micronutrient powders in Bihar, India (*n*=2,170)**

| Characteristic | | Estimate^1^ | |
| --- | --- | --- | --- |
| Households in intervention communities reporting having heard of or seen multiple micronutrient powders (MNP) | | 81.5 | |
| Households reporting having ever received MNP, % | | 70.2 | |
|  | Age when first received MNP^2^, months | 8.0 (7.8, 8.1) | |
|  | Ever consumed MNP^,2,3^, % | 85.2 | |
|  | Received MNP supply in the previous month^2^, % | 25.6 | |
|  | Consumed MNP in the previous month^2^, % | 63.5 | |
|  | Consumed MNP in the previous week^2^, % | 43.0 | |
|  | Currently using MNP^2^, % | 39.4 | |
| ^1^ Values are mean (95% CI) or %. Complex survey procedures used to account for clustering of the data.  ^2^ Percentage out of those who had ever received MNP (*n*=1,523)  ^3^ Only 0.28% of the control group had ever consumed MNP | | |  |

**Supplemental Table 2: Child feeding practices, illness and nutritional status among children 6-18 months** **at end line of cluster randomized controlled effectiveness trial of multiple micronutrient powders in Bihar, India**

| Child nutritional status | Control^1^  (*n*=1406) | Intervention^1^  (*n*=1399) |
| --- | --- | --- |
| Hemoglobin, g/dL | 10.4 (10.4, 10.5) | 10.4 (10.4, 10.5) |
| Anemia^2^, % | 66.1 (62.4, 69.7) | 64.4 (60.8, 68.0) |
| Length-for-age Z-score | -1.4 (-1.5, -1.3) | -1.4 (-1.5, -1.3) |
| Stunted^3^, % | 29.9 (27.3, 32.6) | 27.7 (25.2, 30.2) |
| Weight-for-age Z-score | -1.4 (-1.4, 1.3) | -1.3 (-1.4, -1.3) |
| Underweight^4^, % | 27.8 (25.2, 30.3) | 25.8 (23.2, 28.3) |
| Weight-for-length Z-score | -0.9 (-1.0, -0.8) | -0.8 (-0.9, -0.7) |
| Wasted^5^, % | 13.2 (11.4, 15.1) | 11.7 (9.9, 13.4) |
| IYCF Practices | **Control**^1^  **(*n*=2176)** | **Intervention**^1^  **(*n*=2184)** |
| Currently breastfeeding, % | 94.1 (93.0, 95.2) | 93.3 (92.2, 94.5) |
| Early initiation of breastfeeding (≤1 hour), % | 47.1 (44.0, 50.1) | 47.2 (43.9, 56.1) |
| Avoided giving prelacteal foods, % | 69.9 (67.4, 72.3) | 70.7 (68.1, 73.3) |
| Age of initiation of complementary foods, months | 7.2 (7.1, 7.3) | 7.1 (7.0, 7.2) |
| Timely initiation, % | 46.2 (43.2, 49.2) | 46.0 (43.0, 49.1) |
| Early initiation (<6 mo), % | 4.6 (3.6, 5.6) | 4.5 (3.3, 5.7) |
| Late initiation (>7 mo), % | 49.2 (46.2, 52.2) | 49.5 (46.3, 52.7) |
| Minimum dietary diversity^6^, % | 39.7 (36.9, 42.4) | 39.7 (37.4, 42.0) |
| Minimum meal frequency^7^, % | 75.2 (72.9, 77.5) | 71.7 (69.5, 73.9) |
| Minimum acceptable diet^8^, % | 23.1 (20.9, 25.4) | 22.2 (20.5, 23.9) |
| Child illness (self-reported in past 2 weeks) | **Control**^1^  **(*n*=2176)** | **Intervention**^1^  **(*n*=2184)** |
| Diarrhea^9^, % | 11.1 (9.5, 12.8) | 15.6 (13.6, 17.6) |
| Bloody, % | 4.3 (2.0, 6.6) | 5.4 (2.3, 8.5) |
| Persistent, % | 1.5 (0.2, 2.8) | 3.8 (1.5, 6.0) |
| Severe, % | 66.0 (59.5, 72.4) | 59.0 (51.7, 66.3) |
| Hospitalization, % | 0.2 (0.0, 0.4) | 0.1 (0.0, 0.2) |
| Fever, % | 37.6 (35.3, 40.0) | 34.6 (32.5, 36.7) |

^1^Values are mean or % (95% CI). Complex survey procedures used to account for clustering of the data.

^2^ Hemoglobin concentration <11 g/dL

^3^ Length-for-age z-score <-2;

^4^ Weight-for-age z-score <-2;

^5^ Weight-for-length z-score <-2

^6^ ≥4 food groups from the Child Dietary Diversity Scale

^7^ Two meals for breastfed infants 6–8 months, 3 meals for breastfed children 9–23 months, 4 meals for non-breastfed children 6–23 months

^8^ Minimum dietary diversity and minimum meal frequency

^9^ Bloody diarrhea: presence of blood in stool, persistent diarrhea: >14 days of diarrhea, severe diarrhea: ≥6 loose stools per day during the previous two weeks
